# Are Israelis becoming healthier? Trends in self-rated health, 2002–2018

**DOI:** 10.1186/s13584-020-00409-x

**Published:** 2020-11-26

**Authors:** Anat Ziv, J. Jona Schellekens

**Affiliations:** 1 University of New Brunswick, Fredericton, New Brunswick Canada; 2 Department of Sociology and Anthropology, Faculty of Social Sciences, Hebrew University of Jerusalem, Mount Scopus, 91905 Jerusalem, Israel

**Keywords:** Self-rated health, Education, National expenditure on health

## Abstract

**Background:**

Life expectancy at birth in Israel is steadily increasing. This raises the question whether Israelis are becoming healthier. The purpose of this study is to estimate trends in morbidity and to try to explain what causes morbidity levels to change.

**Methods:**

We used 17 years of repeated cross-sectional data from the Social Survey to estimate trends in self-rated health. We used regression models to explain the trends in self-rated health that were observed from 2002 to 2018.

**Results:**

Four major findings emerged. First, morbidity as measured by self-rated health has declined. Second, gains in educational attainment do not explain the decline in morbidity. Third, the rise in national expenditure on health per capita is strongly correlated with the decline in morbidity. And fourth, the effect of the national expenditure on health per capita appears to be stronger among women and among those without an academic degree.

**Conclusions:**

Self-rated health has improved. However, it has not improved to the same extent for all Israelis. The results of this study show that the health of women has improved more than that of men and that the health of non-academics has improved more than that of academics. The latter suggests that the progressive effect of public financing has offset the regressive effect of out-of-pocket payments on self-rated health.

## Background

Life expectancy in Israel and in other high-income countries has been constantly rising. This raises the question whether gains in longevity are associated with longer periods of morbidity? The results are mixed. Whereas studies in some countries reported a decline in morbidity over time, studies in other countries found no improvement in health or even worsening health over time [1, 2]. Thus, it is not clear what to expect in Israel. This is the first article to present estimates of trends in morbidity in Israel.

Very little is known about what factors influence trends in morbidity. Education is one factor. Adults with higher educational attainments live healthier lives compared with their less educated peers [3]. Education is associated with many health-related behaviors, with access to health care, and with the ability to navigate the health-care system [4, 5]. Education is also associated with psychosocial factors, such as depression, social isolation, stress, and loss of control, which may impact health [6–10]. A recent study has shown that the improvement in self-rated health above age 50 in the United States from 1972 to 2018 is largely explained by gains in educational attainment [11]. But whether this is also true for other countries is unknown. As in the United States, the percentage of academics in Israel has increased substantially.

Medical care is another factor. The debate about the contribution of medical care to the decline in mortality is well known [12]. There is a similar, but lesser-known, debate about the contribution of medical care to the decline in morbidity [13]. The quality of medical care is a function of the national expenditure on health. Thus, the growth in national expenditure on health may be associated with a decline in morbidity [14].

This article has two major aims. First, it will describe trends in morbidity since 2002, when annual data became available. And second, it will try to explain these trends by changes in individual characteristics and in macroeconomic determinants of health, such as the national expenditure on health.

## Methods

### Data and variables

The data for this study was obtained from the Israel Social Survey (ISS), which is an annual cross-sectional face-to-face interview survey of individuals aged 20 and over. The first wave was conducted in 2002. The most recent wave available for analysis dates from 2018. The ISS was designed to provide information on the well-being of the adult population and its living conditions. Each year, the survey consists of two parts: a core questionnaire and a variable module devoted to one or two topics requesting more detailed data than in the core questionnaire. The core questionnaire includes questions on self-rated health and on functional disability.

Low response rates may cause bias. The response rate in the ISS has not declined over time and remains relatively high. In 2002 and in 2018 it was 78% [15, 16]. In 2018 the response rate for the Arabic speaking minority, most of whom are Muslims, was only marginally lower (77%). These response rates are much higher than those in the National Health Interview Survey in the United States, for example, which hover around 60% in more recent years [17].

Self-rated health (SRH) is the outcome variable in our analysis. SRH is based on a single question that asks people to rate their overall health status. SRH follows an ordinal scale: 1 = “not at all good”; 2 = “not so good”; 3 = “good”; and 4 = “very good.” SRH has been found to be very predictive of mortality and strongly correlated with objective assessments of health [18].

**Fig. 1:**
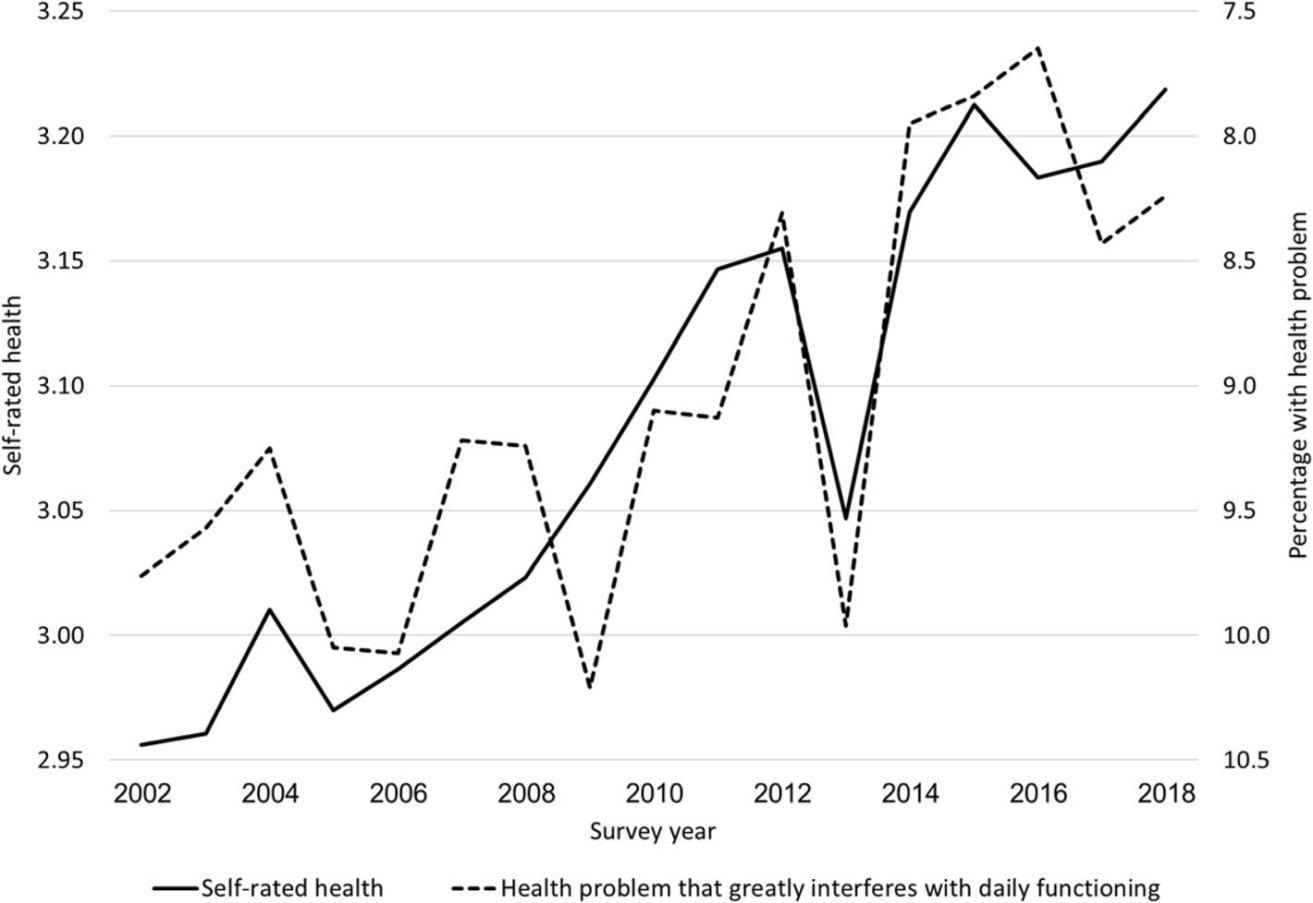
Trends in self-rated health and in the percentage of respondents with a health problem that greatly interferes with daily functioning, Israel 2002–2018. *Note*: Self-rated health follows an ordinal scale: 1 = “not at all good”; 2 = “not so good”; 3 = “good”; and 4 = “very good.” *Source*: Israel Social Survey

The use of SRH may need some justification. The percentage of respondents with a health problem that greatly interferes with daily functioning is a more objective measure of health than SRH. Information on functional disability is also available from the ISS on an annual basis. Figure 1 shows that trends in SRH and in the percentage of respondents with a health problem that greatly interferes with daily functioning are similar. (Note that the values of the right y-axis are in reverse order.) It also shows that trends in functional disability are more erratic, probably because they are based on smaller numbers. Therefore, we will focus on trends in SRH.

Predictors of SRH include individual-level characteristics, such as gender, age, marital status, religion, religiosity, unemployment, and education.

We control for gender, because men are more likely to report being in good health than women [19]. Age is coded as: 30–34, 35–39, 40–44, 45–49, 50–54, 55–59, 60–64, 65–74, and 75 and above. Considerable support has been found for the thesis that marriage is associated with higher levels of SRH [20]. Marital status is coded as single, married, separated or divorced, and widowed.

There may be differences in SRH between religions [21]. Therefore, we added variables indicating the religious affiliation of the respondent. The analysis includes the following religious groups: Jews, Muslims, Christians and Druze. Religiosity has a positive association with SRH [22]. The ISS asked Jewish respondents to define themselves as “Ultra-Orthodox”, “religious”, “traditional but religious”, “traditional but not so religious” or “non-religious/secular”. Differences in SRH between the latter three categories are very small. Therefore, we have combined them into one category, to which we will refer to below as the “non-religious”, for the sake of simplicity. The number of Muslims, Christians and Druze in the sample is relatively small. Therefore, we have not split them by level of religiosity. Non-religious Jews constitute the reference category, which also includes a small number of respondents without religion (2.3%), most of whom are immigrants from the former Soviet Union.

Previous research has shown that unemployment is associated with a low level of SRH [23]. The analysis includes a variable indicating whether the respondent is unemployed. The omitted category includes respondents who were employed and those who were not in the labor force, i.e. respondents who were not employed and did not seek a job, such as housewives and pensioners.

According to the ISS, the percentage of respondents above age 30 with an academic degree has risen by 50 % from 24 in 2002 to 36 in 2018. Education is associated with many health-related behaviors. Thus, better education may explain the decline in the age-standardized percentage of cigarette smokers between 2003 and 2015 [24–26]. The analysis includes a variable indicating whether the respondent has an academic degree. We have limited the analysis to respondents aged 30 and above to give them the opportunity to obtain an academic degree.

There are also macroeconomic determinants of health. For example, the quality of medical care may be a function of the national expenditure on health [27]. Therefore, we have included the national expenditure on health per capita (in 1.000 2015 NIS) as a predictor to the analysis [28].

The pooled sample of the ISS includes 123,838 respondents. However, the analysis was limited to respondents above age 30. Another 274 cases were lost due to non-response on several variables. Thus, the analysis is based on 95,749 cases.

### Analytic methods

The population of Israel is aging. In 2002 16% of the respondents in the ISS were above age 65, whereas in 2018 almost 20% were in this age group. Therefore, we computed age-standardized rather than crude trends in SRH, assuming that the age distribution remained constant at its initial level of 2002.

We used regression models to test hypotheses about the determinants of trends in SRH. Multinomial logistic regression models are recommended, when predicting the probability of category membership on a dependent variable. However, we used linear regression models in order to facilitate the comparison of coefficients across models [29].

To determine the extent to which variables in the regression analysis explain trends in SRH, we computed average predicted values of SRH for each survey year. We used Pearson correlations between the observed and predicted trends in SRH as a measure of goodness of fit for each statistical model.

## Results

**Fig. 2:**
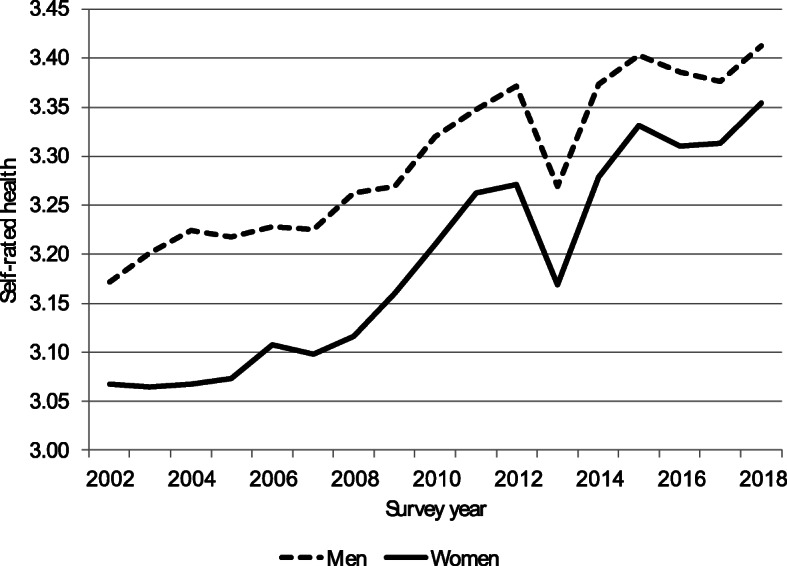
Age-standardized trends in self-rated health by gender, Israel 2002–2018. *Note*: Self-rated health follows an ordinal scale: 1 = “not at all good”; 2 = “not so good”; 3 = “good”; and 4 = “very good.” The standard population used for age-standardization is Israel in 2002

**Fig. 3:**
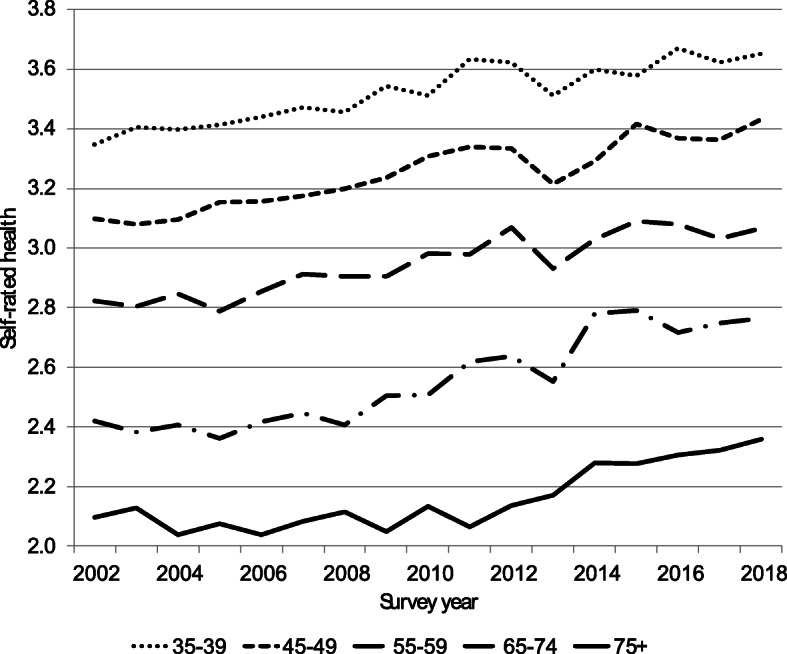
Trends in self-rated health by selected age groups, Israel 2002–2018. *Note*: Self-rated health follows an ordinal scale: 1 = “not at all good”; 2 = “not so good”; 3 = “good”; and 4 = “very good”

Figure 2 shows age-standardized trends in SRH of men (dashed line) and women (solid line). Even though their life expectancy is lower than that of women, men are more likely to report being in good or very good health than women. However, SRH has increased faster among women. Thus, the gender gap has become smaller. Figure 3 shows trends in SRH for selected age groups. It shows that SRH appears to have improved in all age groups.

To answer the question what factors are responsible for the improvement in SRH, Table 1 presents two linear regression models of SRH. The first model only includes individual-level characteristics. The first column in Table 1 presents frequencies of the explanatory variables included in the analysis.

**Table 1:** Linear regression models of self-rated health, Israel 2002–2018

	Percent	Model 1		Model 2	
		***b***	***S.E.***	***b***	***S.E.***
**Gender**					
Female	52.1	−0.077	0.005	−0.305	0.049
Male	47.9	0.000	–	0.000	–
**Age group**					
30–34	14.2	0.000	–	0.000	–
35–39	12.8	−0.101	0.010	−0.105	0.010
40–44	12.0	−0.213	0.010	−0.222	0.010
45–49	10.9	−0.364	0.010	−0.369	0.010
50–54	10.3	−0.522	0.010	−0.525	0.010
55–59	9.6	−0.670	0.011	−0.678	0.011
60–64	8.1	−0.778	0.011	−0.801	0.011
65–74	11.9	−1.015	0.010	−1.036	0.010
75+	10.2	−1.374	0.011	−1.393	0.011
**Marital status**					
Single	8.0	−0.086	0.010	−0.102	0.009
Married	74.6	0.000	–	0.000	–
Divorced or separated	9.5	−0.155	0.009	−0.164	0.009
Widowed	7.9	−0.214	0.011	−0.201	0.011
**Religion and religiosity**					
Non-religious Jew	70.2	0.000	–	0.000	–
Religious Jew	8.2	0.013	0.009	0.007	0.009
Ultra-Orthodox Jew	5.4	0.199	0.011	0.174	0.011
Muslim	11.2	−0.240	0.008	−0.263	0.008
Christian	3.5	−0.232	0.014	−0.238	0.014
Druze	1.5	−0.246	0.021	−0.262	0.021
**Education**					
No academic degree	69.0	0.000	–	0.000	–
Academic Degree	31.0	0.249	0.006	0.442	0.054
**Unemployed**	3.8	−0.122	0.013	−0.104	0.013
**Expenditure on health per capita**	–	–	–	0.087	0.004
Interaction with gender	–	–	–	0.024	0.005
Interaction with academic degree	–	–	–	−0.022	0.006
**Constant**	–	3.635	0.008	2.830	0.039
**R**^**2**^		0.290		0.299	
**Number of cases**	95,749				

*Source*: Social Survey

Married respondents tend to be healthier (see first model in Table 1). Ultra-Orthodox Jews constitute the healthiest religious group, followed by other Jews, Christians, and Muslims, in that order. The difference between religious and non-religious Jews and that between Muslims and the Druze is very small and not statistically significant. The unemployed are less healthy, whereas respondents with an academic degree tend to be healthier than those without an academic degree.

**Fig. 4:**
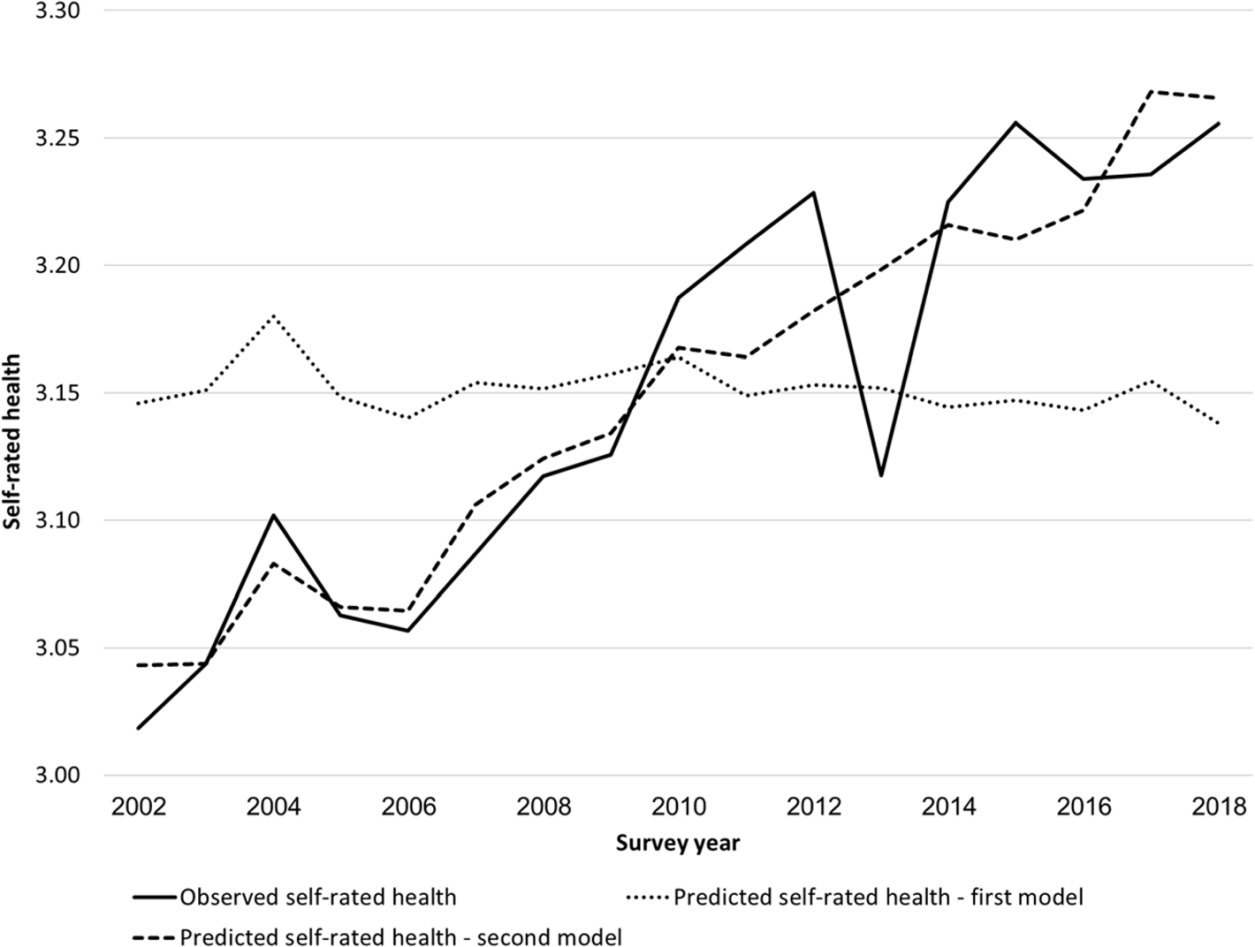
Observed trends in self-rated health and those predicted by a regression model with individual characteristics only and a model with individual characteristics and the national expenditure on health per capita, Israel 2002–2018. Source: Social Survey and coefficients reported in Table 1

The percentage of academics and the percentage of Ultra-Orthodox Jews in the ISS sample have increased, whereas the unemployment rate has declined. To determine whether the correlations detected in the regression analysis are valid over time, we computed average predicted values of SRH for each survey year. Figure 4 compares the observed trend in SRH (solid line) with the trend predicted by the first model (dotted line). It shows that the first model is incapable of explaining the improvement in SRH. Thus, gains in educational attainment do not account for the improvement in SRH. Neither does the rise in the percentage of Ultra-Orthodox Jews nor the decline in the unemployment rate explain the rise in SRH.

The second model in Table 1 adds a macroeconomic determinant of health, the national expenditure on health per capita. Figure 2 shows that SRH has increased faster among women. Therefore, we added an interaction effect between the national expenditure on health per capita and gender. We also added an interaction effect between the national expenditure on health per capita and the variable indicating whether the respondent has an academic degree. Adding the national expenditure on health per capita and its interactions with gender and education only has a marginal effect on the coefficients of the other predictors, except for the variable indicating unemployment.^1^

Table 1 shows that the national expenditure on health per capita has a positive association with SRH. Moreover, the association is stronger among women, whereas it is weaker among respondents with an academic degree, as indicated by the interaction effects. In a preliminary analysis, we also added interaction effects between the national expenditure on health per capita and religious group. However, none of these interaction effects was significant at the level of 5%, suggesting that all religious groups benefited to the same extent from the rise in the national expenditure on health.

Table 1 also shows that the coefficient for the national expenditure on health per capita has a very small standard error, but that does not necessarily imply that it explains much of the rise in SRH. To estimate the contribution of the rise in the national expenditure on health per capita to the rise in SRH, we computed average predicted values of SRH for each survey year. Figure 4 presents the result (dashed line). It shows that the second model is capable of predicting major trends in SRH, except for the sudden drop in SRH in 2013, for which we were unable to find an explanation. The Pearson correlation between the observed trend in SRH and that predicted by the second model is 0.92. Thus, we cannot reject the hypothesis that the rise in the national expenditure on health explains the improvement in SRH.

## Discussion

Life expectancy at birth in Israel is steadily increasing. This raises the question whether Israelis are becoming healthier? Our results indicate that SRH in Israel has improved over the past two decades.

In general, there is much less research on the determinants of trends in SRH than there is on inequalities in SRH. Moreover, studies on inequalities in SRH are not limited to the United States [30, 31]. This study is one of the few to explain trends in SRH and the first to do so outside the United States. Unlike, the United States, we did not find evidence for the contribution of gains in educational attainment. However, the hypothesis that the quality of medical care is a function of national expenditure on health fits the Israeli data. Moreover, it is able to account for most of the rise in SRH.

Our results show that women are catching up with men and that individuals without an academic degree are catching up with those with an academic degree. If there is a causal relationship between the rise in the national expenditure on health per capita and the decline in morbidity, then the stronger effect of the national expenditure on health per capita among those without an academic degree suggests that the progressive effect of public financing has offset the regressive effect of out-of-pocket payments on self-rated health [32].

A major strength of this study is that its results are based on a very large sample. However, there are also limitations. For example, we were unable to find an explanation for the sudden drop in SRH in 2013. The drop has been observed in all age groups, except for 75+ (see Fig. 3). There also was a rise in the percentage of respondents with a health problem that greatly interferes with daily functioning (see Fig. 1). A similar drop occurred in overall life satisfaction (OLS).

A more important limitation of the current study is that all analyses were correlational. We found that there is a strong correlation between trends in SRH and those in the national expenditure on health per capita. The second model explains 85% of the trends in SRH (*p*-value < 0.01). If we omit the outlier of 2013, then the percentage explained even rises to 91. However, our results do not show the direction of causality. Moreover, we cannot rule out the possibility that an omitted variable caused the decline in morbidity. However, trends in such a variable would be strongly correlated with trends in the national expenditure on health per capita, because the second model explains most of the decline in morbidity.

Previous research has shown that there is a strong association between SRH and OLS. This is also true for Israel [33]. Moreover, both measures show similar trends. Of course, it is possible that the improvement in SRH explains the rise in OLS. However, we cannot rule out the possibility that the rise in OLS contributed to the improvement in SRH [34]. Because of the possibility of reverse causality, we did not include OLS as an explanatory variable in the analysis. If trends in OLS explain those in SRH, then we are left with the question what explains trends in OLS. It is possible that the rise in living standards, as measured by the gross domestic product (GDP) per capita, drives trends in OLS. The national expenditure on health is nearly constant as a percentage of GDP per capita. Thus, the rise in living standards may drive trends in SRH either through the national expenditure on health or through OLS.

If trends in the national expenditure on health drive trends in SRH, then our results may have policy implications. Our statistical model predicts that an additional rise in the national expenditure on health will improve SRH even more.

## Conclusions

To the best of our knowledge, there are no previous studies of trends in SRH in Israel. Four major findings emerged. First, there has not been an expansion of morbidity in the last two decades in Israel. Second, gains in educational attainment do not explain the decline in morbidity. Third, trends in SRH are correlated with trends in the national expenditure on health per capita. And fourth, some appear to have benefited more than others from the rise in the national expenditure on health per capita, such as women and those without an academic degree.

## Data Availability

Data available from the Israel Central Bureau of Statistics.

## Abbreviations

ISS: Israel Social Survey
OLS: Overall life satisfaction
SRH: Self-rated health

## Footnotes

1 We have tested the assumption that the associations between the independent variables and SRH do not change over time by adding interactions between all the independent variables and the annual expenditure on health per capita, as a proxy for the time trend. The interactions with gender, education and the variable indicating whether the respondent is separated or divorced are relatively large compared with their standard error, but not those with most age groups, the religious variables and unemployment. Therefore the final results in Table 1 only include the interactions with gender and education. Thus, we have omitted the interactions with age groups, the religious variables, unemployment and marital status. We have omitted the latter, because only the interaction with the variable indicating whether the respondent is separated or divorced is relatively large.
